# Report of the Condition of the Sixth Health District of Buffalo

**Published:** 1892-10

**Authors:** A. L. Benedict


### REPORT OF THE CONDITION OF THE SIXTH HEALTH DISTRICT OF BUFFALO.

By A. L. BENEDICT, M. D.

For sanitary purposes the sixth district can be best considered in sections, as follows :

Section 1. Bounded by Front avenue, Wilkeson, Mohawk, and Niagara streets, Delaware avenue, Church, and South Division streets, Washington street, East Tupper and Michigan streets, Genesee, Jefferson, Virginia, Cottage, and Hudson streets.

Sec. 2. Bounded by West avenue, Hudson street, the lake and river, Georgia, and Court streets.

Sec. 3. Bounded by Georgia and Court streets, the Terrace, Erie street, the water front.

Sec. 4. Between Main and Michigan streets, the Hamburg Canal, and Seneca streets.

Sec. 5. Between Swan and Genesee, Washington and Michigan streets.

The sanitary condition of the first section is generally good. There is an abundance of vegetation and of "breathing spaces." The streets are, for the most part, well paved, and many are asphalted. Most of the dwellings are occupied by only one family, and crowding occurs only in blocks along some of the business streets. Privy vaults are not numerous, except in the eastern and extreme western portions of the section. The inhabitants can hardly be approached by a sanitary official, but they will probably avail themselves of suggestions published in the newspapers.

Section 2 contains a few tenement houses. Most of the dwellings are cottages occupied by one family, or two-story houses occupied by two or three families. On account of the large number of children, and the carelessness rather than poverty of the adults, there is much sickness. While this section is not closely built up, there is little vegetation to aid in the oxidation of organic matter. West of the canal, sanitary provisions are conspicuous by their absence. Privy vaults are necessities, and the canal water is used for domestic purposes. An abundance of fresh air is, however, a favorable feature.

Section 3 contains a number of fairly good tenement houses and rows of smaller houses, as on Mechanic and Barker streets. The proximity of the canal and slips, the comparative lack of vegetation, a number of filthy privy vaults, and of unpaved streets, into which garbage is thrown, tend to make this section unwholesome. Many of the inhabitants are Italians, who cannot understand English. They are, however, tractable and obedient patients, and are much more cleanly in their habits than is generally supposed. They are prone to eat stale meat and fruits, and, on account of their gregarious habits, an infectious disease would spread rapidly among them.

Section 4 comprises part of the real tenement house district of Buffalo. In the same building may be found people of various nationalities, representing various degrees of comfort and of squalor. Most of the buildings have water-closets, some in good order, many in a filthy condition. This part of the city is very compactly built, and there is much sickness.

Section 5 is in a transition state. Many families are in comfortable and sanitary quarters, others are crowded and surrounded by dirt.

Special attention is called to the following topics:

*Livery stables* are numerous in the sixth health district, and are, for the most part, close to residences. To prevent fermentation and putrefaction, manure should be removed daily. Disinfection of the stable floors would be easy and cheap.

*Saloons, Restaurants, Small Hotels.*—The water-closets or privies of these places are frequently extremely filthy. The food supplied to customers is apt to be of bad quality. The discharges from the stomach and bowels of drunken men, left in rooms without attendance, may infect floors, walls, furniture and bedding. The proximity of the depots to the Exchange street restaurants will make them points of suspicion if cholera gains a foothold in this country.

*Railroads.*—Very fortunately the freight and passenger depots prevent the too close approach of dwellings to a long stretch of the Hamburg Canal. Most of the water-closets in the depots are in fair condition. The Central tracks run through the sixth health district. As railroads are the principal carriers of infection to inland towns, and as the main exit of the cholera spirillum is through the intestines, the closets in passenger cars should be kept locked inside the city limits. In fact, it would be better, though more expensive and troublesome, to have a metal bucket fitted to the ordinary open cylinder of the car closet. A disinfectant could be kept in the bucket, which could be changed at certain stations and the disinfection completed.

*Business Blocks.*—There are many in the sixth district. In some of them a large force of men are employed and the closets are in a filthy condition. As physicians are rarely called to these buildings, the sanitation can only be determined by special inspectors.

*Churches.*—Vaults should be inspected and disinfected if necessary. If cholera comes, the vaults should not be used this winter. Regulations with regard to funerals will, of course, be enforced. The clergy, of the catholic church especially, could do much to instruct the people in simple hygienic rules. Entirely aside from the possible danger of cholera, it must be recollected that a large proportion of deaths in childhood occur from too frequent nursing and from improper feeding of older children. These evils cannot be guarded against by physicians except when much harm has already been done. The influence of an intelligent priesthood could reach a large number of persons, especially foreigners, who cannot receive instruction through newspapers and bulletins. If cholera becomes general, church meetings and all public assemblies should be discouraged.

*Pavements and New Buildings.*—Disturbance of the soil should be avoided as far as possible. Cellar digging, even in the best sections of the city, is apt to upturn the contents of old privy vaults which have been filled in without having been emptied.

Garbage should be gathered three times a week for at least another month. Collections should be made in the day time, so that the odor would give warning and afford an opportunity to close doors and windows. Asphalt streets should be washed with streams of water from fire hose after the garbage has been collected. Possibly, even stone pavements would stand the force of the water. Street sprinklers are an abomination, as they serve only to furnish the moisture necessary to convert the dirt into a nidus for germs. Clean streets need no sprinkling to "lay the dust," and the only way in which sprinklers should be allowed are on dirt roads and in advance of street sweepers.

*Water.*—All efforts to economize water should be abandoned, and people should be encouraged to flush out drains and sinks daily.

*Dead bodies* of cholera patients should be cremated. Popular prejudice would, however, probably not allow the enforcement of this principle. Thorough arterial and cavity embalming should, at least, be insisted upon.

*Markets, grocery stores, and butcher shops* are numerous in the sixth district. Decayed vegetables, fruit, and meat should be frequently removed, especially from cellars. Venders of fruit from stands and wagons are particularly apt to carry a stale stock of fruit and vegetables. Licenses should be granted with caution, and inspection should be adequate. The Chippewa market should be carefully attended to, and the whole area should be cleaned and disinfected after market hours. Being paved with stone blocks, the out-door market has no safeguard against infection by soakage. It is not probable, however, that any specific choleraic focus will be established here, unless it starts from the public closets, whose condition is far from perfect.

It is believed by the writer that the preceding report, hastily prepared, will give a general idea of the condition of the sixth health district, and of some of the special dangers to be guarded against in it. Incidentally, some more general suggestions have been introduced, and in them no effort has been made to distinguish between those relating to ordinary sanitary measures and those aiming particularly against cholera Asiatica.

---

THE Health Commissioner of Buffalo has issued the following circulars :

[*Official Health Bulletin No. 1.*]

### PRECAUTIONS AGAINST CHOLERA.

In view of the possibility of a visitation of Asiatic cholera, the Department of Health calls the attention of householders and others whom it may concern, to the importance and necessity of placing their premises and surroundings in a thoroughly sanitary condition. With this object, a careful perusal and compliance with the following is officially urged :

Water-closets should be kept scrupulously clean, frequently flushed and disinfected. Privy vaults, cesspools, and other nasty substitutes for excreta are a menace to health at all times. Under the present circumstances, therefore, they demand the utmost attention, and should be kept particularly clean, ventilated, and disinfected, and additionally all communicating sewers thereto should be kept well flushed, freed from defects, and likewise disinfected.

Drain pipes, sinks, and all channels in which refuge is carried off, should be inspected, repaired where necessary, kept free, frequently flushed and disinfected.

The emptying of slops, the spilling of garbage, or the accumulation of refuse upon the surface, either around the premises or in adjacent alleys, is dangerous in the extreme, and is imperatively forbidden. House refuse while awaiting removal should be confined in covered metallic receptacles and frequently disinfected. The burning of garbage is the best method of its disposal, and is recommended.

Particular attention is directed to dark and damp cellars, with their possible accumulation of filth therein. Such should be promptly cleaned, well ventilated, and chloride of lime freely used.

Well and cistern water being most liable to pollution, their surroundings should be especially looked after and any and all sources of contamination attended to.

The attention of those engaged in the so-called offensive trades, as well as keepers of stables, slaughter houses, and other similar places, is particularly called to the importance of the subject, and, in view of the impending danger, are directed to immediately put their premises in such condition of cleanliness that germs will find no congenial soil.

Among the readily obtainable disinfectants for common use may be mentioned chloride of lime, which is cheap, but must be employed freely and often. It is applicable for privy vaults, cellars, stables, etc.

Carbolic acid in the proportion of one part to twenty-five, or corrosive sublimate, one part to 500, are adapted to surface disinfection.

Copperas, one pound to a bucket full of water, may be employed in out-houses, sinks, and drains.

Chlorinated lime may be said to be the best germicide for the purification of filth of all descriptions.

The whitewashing of out-buildings, cellars, stables, places liable to harbor dirt and attract infection, is one of the cheapest and best means of maintaining them in a cleanly condition.

ERNEST WENDE,
*Health Commissioner.*

---

[*Official Health Bulletin No. 2.*]

### TENEMENT HOUSES.

One of the true safeguards against the visitation of Asiatic cholera is the general cleansing and disinfection beforehand. Therefore, it is directed that owners and agents of tenement houses will forthwith place their respective buildings and their surroundings in the best sanitary condition. This implies an inspection and correction of all offensive conditions from cellar to garret.

Cellars must be ventilated, thoroughly cleaned, and all accumulation of refuse from corners and other obscure places promptly removed therefrom.

Further, the free use of chloride of lime to the floors, the cleansing and whitewashing of the walls with more than one coat, and the placing of boxes of quicklime in suitable places, purifies and dries the air, and renders the cellar wholesome.

Walls throughout the buildings, especially in overcrowded rooms, the halls, and dark passages, can be placed in a cleanly condition by the free use of whitewash.

Drains, sinks, water-closets, and other vehicles for the removal of waste and refuse, must be put in repair, cleansed, and from time to time treated with an effective disinfecting solution.

The grounds and surroundings, so often, through carelessness, dirty and littered with offending material, must be cleaned and kept undefiled.

Outdoor privies must likewise be cleansed, kept so, and disinfectants freely used in and around them.

In this connection, attention is called to the fact that, upon failure of those responsible in the matter, the Department of Health is empowered by law to make sanitary any condition detrimental to the public welfare, the cost of the same being a lien upon the property.

ERNEST WENDE,
*Health Commissioner.*

---

[*Official Health Bulletin No. 3.*]

### CARE OF THE PERSON IN RELATION TO CHOLERA.

The liability to infection from pestilential diseases, more especially Asiatic cholera, can be materially diminished by the observation of certain laws of health and habit. The testimony of history bears this fact out to the fullest extent. Inasmuch as the disease assails only by the way of the intestinal canal, the indications are largely to maintain this portion of the body up to the best standard by guarding it against all sources of irritation and contamination, and correcting all deviations and slight ailments of it without delay.

The following suggestions in relation thereto are made for the benefit of the public with the fullest confidence :

The infecting cholera germs being destroyed by heat, and most likely to be introduced through articles of food and drink, such should receive the closest scrutiny as to the condition and cleanliness, and to be taken only in a cooked form.

Water should be boiled, and milk, from its great absorbing properties, likewise treated and kept in the most cleanly of places, essentially so where its use is intended for children.

Fruits and all other raw articles of food should be avoided, as they are liable to excite diarrheal troubles, and are prone to accidental contamination, while canned goods, from their mode of preparation, hold an important place. Pains should be taken to keep kitchen utensils, tableware, etc., absolutely clean, and in the advent of disease being present, should be scalded before using.

Cleanliness of the person is more important, if possible, than ever. The use of alcohol should be avoided, dissipation, excesses of all kinds abandoned, and not the least in eating. Disturbances of the bowels and stomach increase the liability to such a marked degree, that the apparently most simple irregularity is sufficient justification to immediately obtain medical advice, which, with our equipment of dispensaries and hospitals, is available as well to the poor.

During the prevalence of the disease, these precautions become imperative. In households, which may be visited by the malady, additional care becomes necessary, and the instructions of the attending physician followed faithfully. It is sufficient here to refer to the fact that the contagion exists only in the stools and vomited matter. Therefore, furniture, clothing, bedding, utensils, as well as the hands and person, contain and carry the infection should they become soiled in the slightest manner. In such event they must be at once cleansed and disinfected.

Particular caution is necessary in dealing with the stools. A disinfectant should be added to them as soon as passed; they should be conveyed, covered, and the place where emptied flushed and well disinfected.

Personal fear is to be discouraged, and is in no way warranted with the existing means of dealing with epidemics, and individual common sense and care, danger is reduced to a minimum.

ERNEST WENDE,
*Health Commissioner.*

---

THE metric system is frequently used in prescriptions, pedantically and otherwise, and for the benefit of those not accustomed to this plan, we will insert in this column, in each issue of the *Journal*, the following correct table of metric weight and measure:

The metric unit of fluid measure is the liter, the cube of one-tenth meter, or 1,000 cubic centimeters—equal to about thirty-four fluid ounces. The metric unit of weight is the gram, which represents the weight of one cubic centimeter of water at its maximum destiny. It is equal to about fifteen grains.

One cubic centimeter is equal to about sixteen minims:

| | | | | |
|---|---|---|---|---|
| Thus: | 1-64th | grain=100th | gram, | or 0.001 |
| " | 15 | grs. =1 | gram, | or 1.000 |
| " | 1 | grain=1-15 | gram, | or 0.066 |

By a little study, the above will clear up trouble in reading this system, which is already confusing our readers instead of helping them.—*Hot Springs Medical Journal.*

